# General and Specific Considerations as to why Osteoporosis-Related Care Is Often Suboptimal

**DOI:** 10.1007/s11914-020-00566-7

**Published:** 2020-02-26

**Authors:** Elizabeth M Curtis, Stephen Woolford, Claire Holmes, Cyrus Cooper, Nicholas C Harvey

**Affiliations:** 1 MRC Lifecourse Epidemiology Unit, Southampton General Hospital, University of Southampton, Southampton, UK; 2 Rheumatology Department, University Hospitals Southampton NHS Foundation Trust, Southampton, UK; 3 NIHR Oxford Biomedical Research Unit, University of Oxford, Oxford, UK; 4 NIHR Southampton Biomedical Research Centre, University Hospitals Southampton NHS Foundation Trust, University of Southampton, Southampton, UK

**Keywords:** Osteoporosis, Epidemiology, Fracture, Adverse effects, Treatment gap, Policy

## Abstract

**Purpose of Review:**

The assessment of fracture risk and use of antiosteoporosis medications have increased greatly over the last 20–30 years. However, despite this, osteoporosis care remains suboptimal worldwide. Even in patients who have sustained a fragility fracture, fewer than 20% actually receive appropriate antiosteoporosis therapy in the year following the fracture. There is also evidence that treatment rates have declined substantially in the last 5–10 years, in many countries. The goal of this article is to consider the causes for this decline and consider how this situation could be remedied.

**Recent Findings:**

A number of possible reasons, including the lack of prioritisation of osteoporosis therapy in ageing populations with multimorbidity, disproportionate concerns regarding the rare side effects of anti-resorptives and adverse changes in reimbursement in the USA, have been identified as contributing factors in poor osteoporosis care.

**Summary:**

Improved secondary prevention strategies; screening measures (primary prevention) and appropriate, cost-effective guideline and treatment threshold development could support the optimisation of osteoporosis care and prevention of future fractures.

## Introduction

The management of osteoporosis—its diagnosis, the assessment of fracture risk, the development of therapies and best practice guidelines to reduce the risk of fractures—has advanced hugely over the past 30 years. However, many studies indicate that the care of people with osteoporosis is still not optimal; indeed, most men and women at high fracture risk do not receive treatment [1]. This is true even in patients who have sustained a fragility fracture, with, in many cases, fewer than 20% actually receiving therapies to reduce the risk of fracture in the year following the fracture [2, 3]. Rates of treatment are particularly poor for older women and those living in long-term care. Large gaps in service provision exist, as indicated by the fact that the use of fracture risk assessment tools such as FRAX® varies one thousand-fold worldwide. This variability is far greater than the 30-fold variation in crude, or 10-fold variation in age-standardised hip fracture incidence globally [4, 5]. Global differences in availability of internet access, availability of national assessment guidelines for osteoporosis in many countries and the availability of alternative assessment algorithms only partially explain these differences [4].

Whilst the under-assessment and treatment of those at very high risk of further fracture is of great concern, even more alarming is the apparent downward trend in treatment with antiosteoporosis medications after hip fracture, which has been demonstrated both in the US, European and UK populations [6, 7]. There are many potential causes for this trend, including the elevation of concerns regarding rare antiresorptive drug-related side effects such as atypical femoral fractures and osteonecrosis of the jaw and the recent changes to treatment reimbursement in the USA. In this review, we give an overview of the reasons for suboptimal osteoporosis care, or “treatment gaps”, at all levels, and discuss possible approaches to remedy this problem.

## What Do We Mean by “Suboptimal” Osteoporosis Care?

It is widely recognised that disparities exist between the population at high fracture risk, or who have experienced a low trauma fracture, and the number who receive appropriate osteoporosis assessment and treatment [1]. As an example, in the UK, analysis of the Clinical Practice Research Datalink (CPRD) has demonstrated inadequacies in both primary and secondary fracture prevention. Initial improvements in prescription rates were encouraging; following a hip fracture in the UK, the probability of antiosteoporosis drug prescription increased from just 7% in 2000 to 46% in 2010 [8], with older patients (≥ 75 years of age) particularly benefiting from this trend. At any given point in time, the cumulative incidence of antiosteoporosis therapy was greater in women (8% in 2000, 51% in 2010) than in men (4% in 2000, 34% in 2010). Given this steady increase in the awareness of the need for antiosteoporosis drugs over a 10-year period, a continued trend in increased prescribing would have made perfect sense. However, despite fewer than 50% of hip fracture patients receiving treatment, from around 2011, a plateau and a possible decrease in prescriptions has occurred in the UK [9•]. Local trends in prescribing antiosteoporosis therapies appeared to persist over time, with substantial geographic heterogeneity within the UK, for example, in the North East of the UK the odds ratio for antiosteoporosis medication following a hip fracture was 1.29 (95% CI 0.89, 1.87) and in the South Central region 0.56 (95% CI 0.43, 0.73), the North West being the referent. Over the subsequent 5 years of follow-up, these geographic trends persisted indicating systematic differences in provision of osteoporosis care [10].

Such patterns in secondary fracture prevention are present in many countries. A prospective observational study of over 60,000 older women recruited from primary care practices in 10 countries across USA, Europe and Australia (the GLOW study) demonstrated that over 80% of women with a fragility fracture did not receive osteoporosis treatment [11]. In another international prospective study of 1795 patients who sustained a low trauma hip fractures in ten countries (Australia, Austria, Estonia, France, Italy, Lithuania, Mexico, Russia, Spain, and the UK), just 27% were prescribed antiosteoporosis therapy after the hip fracture [12].

**Fig. 1:**
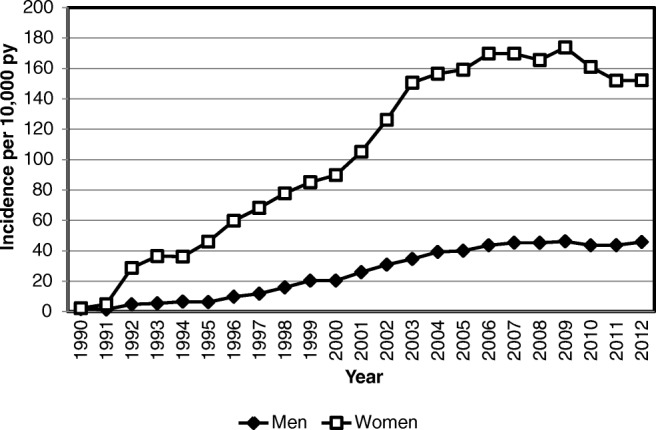
Antiosteoporosis medication prescription incidence from 1990 to 2012 in the UK population aged 50 years or over, reproduced with permission from van der Velde et al. [13]

Trends in prescribing (incorporating both primary and secondary prevention) are consistent with these findings. Figure 1 shows that antiosteoporosis medication prescription rates in the UK rose from 2.3 to 169.7 prescriptions per 10,000 person-years amongst women from 1990 to 2006. However, following this rise, prescription rates plateaued and then decreased by 12% between 2009 and 2012 (Fig. 1). In men, prescription rates rose less steeply from 1990 to 2007 and then plateaued from 2008 onwards, with marked differences in prescription of antiosteoporosis medications according to ethnicity and geographic location in both sexes [13].

**Fig. 2:**
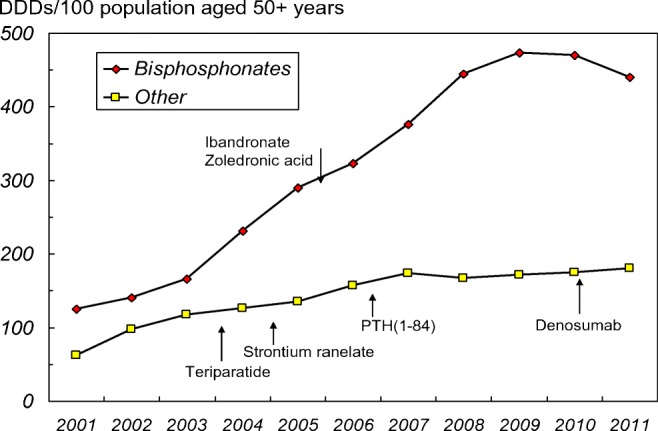
Estimated sales of antiosteoporosis drugs (Defined Daily Doses, DDD)/100 population aged 50 + years from 2001 to 2011 in the European Union, reproduced with permission from Hernlund et al. [14]

Treatment uptake for osteoporosis similarly increased in Europe up to 2008, thereafter plateaued, and in more recent years has fallen (Fig. 2). Within Europe, there is marked inter-country variation in the treatment penetration of individuals at high risk of osteoporotic fracture—in a recent study the percentage of patients at high fracture risk (defined using FRAX®) who were not treated (also known as the treatment gap) varied from just 25% in Spain to 95% in Bulgaria. It was estimated that, within the EU in 2010, out of the 21.3 million men and women who exceeded FRAX high risk level, 12.3 million were untreated [12, 14–17]. These estimates are conservative (since treatment will have been given to an unknown proportion of low risk women) [18], though have been supported by findings from Spanish studies using individual-level data from primary care prescription databases from over 1.5 million participants [19]. In Danish registry studies, osteoporosis medication use plateaued in 2014 and declined thereafter. Interestingly, hip fracture rates also declined between 2005 and 2015 by 30%, but only 20% (at most) of the observed reduction could be attributed to treatment. The authors concluded that the anti-osteoporosis therapy use was at too low a level to make a meaningful impact on the fracture burden within Danish society and represented a missed opportunity for reducing hip fracture rates [20].

Similar findings have emerged from a large retrospective analysis using US administrative insurance claims data of almost 100,000 men and women aged over 50 years who were hospitalised for hip fracture [6]. The study examined the uptake of osteoporosis medication within a year of hospital discharge; the estimated probability of receiving osteoporosis medication within this time period was 28.5% but showed a significant decline over a 10-year interval, from 40.2% in 2002 to 20.5% in 2011 [6]. Findings from the US Medical Expenditure Panel Survey are supportive of this, demonstrating a marked reduction in the prevalence of bisphosphonate use from 2007 onwards, particularly amongst women [21].

## Why Is there a “Treatment Gap”—From Best Practice to Current Practice?

### Awareness and Perception by Patients and Physicians

As shown by the studies cited above, antiresorptive treatment rates both in primary and secondary fracture prevention increased until 5 to 10 years ago. The clinical situation was bolstered by policy and risk assessment advances, including the use of FRAX® and other risk calculators [22, 23] and guidance on intervention [15], combined with the availability of cheaper, generic bisphosphonates. In spite of this, outwardly prospering field treatment rates have declined in recent years, both in those at high risk of fracture and in those who have suffered a fracture, despite the huge expansion of the at-risk population [24].

Many factors appear to contribute to this phenomenon. One such reason is that strategies at a national and international level have not been implemented sufficiently to impact primary and secondary preventions. For patients and clinicians alike, the idea of managing a future “risk” makes primary fracture prevention more difficult, rather than treating a disease event which has already impacted upon the patient. Musculoskeletal diseases in general are viewed by policymakers and patients alike as a of lesser importance than outcomes such as cancers and heart disease [1], despite the fact that the musculoskeletal disease has been shown to be a leading course of disability worldwide by the Global Burden of Disease initiative [25].

In the case of osteoporosis, there is a stark mismatch between the perceived and actual severity of the condition. Many do not recognise that, for example, a hip fracture is associated with a 20% associated reduced survival compared with non-fracture peers and as such is a devastating life event [26]. Contrast this with a parallel event such as an acute myocardial infarction; it would be impossible to imagine that it would be acceptable in the developed world for less than 50% of such cardiac patients to receive risk-reducing therapies such as aspirin and other antiplatelet agents, antihypertensives and statins [27]. The large international women’s cohort, GLOW, clearly documents this risk misperception, in which many underestimated their own fracture risk in comparison with their peers [28]. Perhaps, in a world where many populations are ageing and physicians and patients are dealing with multimorbidity, osteoporosis treatment falls to the bottom of the priority list.

Physicians’ perceptions of osteoporosis and efficacy of treatments have been further confused by harmful and inaccurate conclusions about the treatment of osteoporosis in high impact journals including the British Medical Journal and Journal of Internal Medicine [29, 30]. These articles, claiming, for example that “the dominant approach to hip fracture prevention is neither viable as a public health strategy nor cost effective” and that “the main ways to prevent these fractures have not changed in nearly 25 years: stop smoking, be active and eat well” are frankly incorrect, unbalanced and refuted by overwhelming evidence (as stated by international and national societies such as the International Osteoporosis Foundation and the American Society for Bone and Mineral Research); nonetheless, such “fake news” presented in high impact journals has traction, and clear damage has been done [31].

### Concerns Regarding Medication Adverse Effects

There are abundant data showing that alarming reports about osteoporosis medication in the media have been followed by a reduction in use of these medications, despite evidence that the benefits of treatment clearly outweigh the risks for the vast majority of users [32]. In order to better understand patients’ concerns regarding medication safety, Jha et al. used data from the Medical Expenditure Panel Survey and National Inpatient Sample in the USA to examine relationships between medication use, internet searches for alendronate between 2006 and 2010, and safety concerns reported in the media [21]. Clear spikes of internet search activity were observed to correspond to events such as a 2006 lawsuit filed against Merck (for Fosamax allegedly causing osteonecrosis of the jaw (ONJ)), an ABC World News feature in 2010 on the associations between Fosamax and atypical femoral fractures (AFFs), and various other reports in the media of serious but rare side effects, set in parallel with the regression in bisphosphonate use by more than 50% between 2008 and 2012. The Australian Longitudinal Study on Women’s Health findings was in keeping with the findings from the USA; antiosteoporosis medication use grew over the period 2000 to 2007 but then shrank from 2007 to 2010. In Australia, interventions to remedy this, including the relaxation of the indications for bone density testing and a subsidy for antiosteoporosis medications, had little effect, the most marked declines in prescriptions coincided with negative press for antiresorptive therapy such as a 2007 major report on ONJ [33].

In absolute terms, the serious long-term adverse side effects of bisphosphonates are very rare (with incidences in ranging from 1/100,000 to 1/10,000 per year) [34]. However, the approach to risk/benefit communication has largely been on the side of declaring risk, amongst the media (as demonstrated above), physicians and policymakers. The substantial impact of the underlying condition on morbidity and increased mortality, with fracture risk markedly decreased by antiosteoporosis medications, appears often forgotten in these discussions. For example, UK National Institute for Health and Care Excellence (NICE) recently issued guidance on multi-morbidity [35] and targeted bisphosphonates for review after 3-year treatment despite the evidence for their longer-term efficacy and safety being of greater reliability than the other treatments considered for 3-year review.

In a recent review of the benefits versus risks for BP therapy [36], the benefits for fracture reduction for short-term therapy for 3 to 5 years was shown to far outweigh any risks of AFFs. Using the set of assumptions about AFF risk with best evidence (relative risk of 1.7 for any BP use [37]), the study states that treating 10,000 osteoporotic women for 3 years would lead to the prevention of 1000 fractures (including 110 hip fractures) whilst causing only 0.08 AFFs. Put another way, for one AFF associated with 3 years of BP treatment, 1200 fractures (including about 130 hip and 850 vertebral fractures) would be prevented [38•].

In longer-term users, the concerns regarding the rare side effects of AFFs and ONJ are compounded by studies suggesting that longer therapy duration increases these risks. This has led to the widely held opinion that all patients on long-term treatment with bisphosphonates or denosumab should be offered a treatment holiday; however, the existing evidence does not entirely support this. For example, following denosumab discontinuation, rapid bone loss has been described, with around a 5% incidence of vertebral fractures [39], indicating that treatment holidays on denosumab should not be offered without replacing with another anti-osteoporosis therapy. In terms of long-term bisphosphonate use, reassuringly, a Danish study has demonstrated that users of alendronate, even after 10 years of use, have a lasting reduction in fracture risk compared with matched controls, and that the number of hip fractures prevented is still substantially greater than the number of subtrochanteric femoral fractures occurring [40]. A new systematic review led by the International Osteoporosis Foundation has concluded that drug holidays should only be considered in patients at low fracture risk [41••]. Thus, it is evident that the osteoporosis field needs to vastly improve its approach to communicating the balance of treatment risks and benefits and to work towards countering poorly evidenced stories in the media as and when they occur.

### Policies in Healthcare and Osteoporosis Assessment

Osteoporosis, when compared with other non-communicable diseases, has rarely attracted justifiable levels of attention from governments, policy makers and healthcare providers. National policies on access to and reimbursement of measurement of bone density with dual-energy x-ray absorptiometry (DXA) will have a great impact on the assessment and treatment of this disease. Various regional audits have been published by the International Osteoporosis Foundation (IOF) (https://www.iofbonehealth.org/regional-audits) covering Latin America, the European Union, Eastern Europe the Middle East, Africa, Central Asia and Asia Pacific. These have demonstrated large variations in terms of the epidemiology, financial and societal costs, and burden of osteoporosis e.g. in the Asia Pacific region, whilst Hong Kong, Japan, Republic of Korea, Singapore, Australia and New Zealand had 12–24 DXA machines per million of population, India, Pakistan, China, Indonesia, Philippines, Sri Lanka and Vietnam were severely under-resourced, having less than 1 DXA machine per million of population. The audits demonstrated that insurance or healthcare policies in many countries did not reimburse BMD testing and osteoporosis treatment which served as a barrier to osteoporosis care access. Similar inequalities were seen in Europe, where it was calculated that 11 DXA machines per million of population were needed for adequate osteoporosis care provision. Only 16 European countries fell into this category of “adequate” provision, and 9 countries were considered to have “very inadequate” provision with fewer than 8.4 DXA units per million (the UK, Luxembourg, Czech Republic, Hungary, Bulgaria, Latvia, Lithuania, Poland, Romania and), as shown in Table 1 [42]. In addition, reimbursement for DXA scanning was extremely variable between EU member states, with reimbursement for DXA only offered if the BMD turned out to show osteoporosis in certain countries (Bulgaria and Switzerland), only if BMD was measured after a fracture (Germany), or only if the patient was referred by a specialist (Poland).

**Table 1:** Central DXA units per million of the general population available in the EU27 countries. Adapted with permission from [42]

Country	DXA units/million	Country	DXA units/million	Country	DXA units/million
Austria	28.7	GERMANY	21.1	NETHERLANDS	10.7
Belgium	53.0	GREECE	37.5	POLAND	4.3
Bulgaria	1.2	HUNGARY	6.0	PORTUGAL	26.9
Cyprus	23.9	IRELAND	10.0	ROMANIA	2.4
Czech Republic	5.2	ITALY	18.6	SLOVAKIA	10.7
Denmark	14.6	LATVIA	4.9	SLOVENIA	27.1
Estonia	8.9	LITHUANIA	3.4	SPAIN	8.4
Finland	16.8	LUXEMBURG	2.0	SWEDEN	10.0
France	29.1	MALTA	9.7	UK	8.2

Whilst no official IOF audit is available for North America, treatment reimbursement also varies greatly, depending on each patient’s health insurance. The evolution of healthcare reform in the USA from a “fee for service” system to a system which supports improved disease prevention and care coordination, with financial incentives to encourage healthcare professionals or systems to improve patient outcomes, ought to improve osteoporosis detection and treatment. However, the number of DXA providers has fallen following a major drop in reimbursement in this area, resulting in over 1 million fewer DXA scans performed per year in women aged 50–64 in recent years [43], a change which coincides with a plateau in the secular decline (up until 2012) in age- and sex-adjusted hip fracture rates [44].

## What Can We Do to Optimise Osteoporosis Care?

### Secondary Prevention: Treating Those Who Have Already Had a Fracture

As described by the evidence detailed above, fragility fractures represent a huge burden on societies worldwide. Patient perception of fracture risk is often underestimated as osteoporosis is a silent condition until a fracture happens [45, 46], so primary prevention initiation is usually reliant on health care practitioners who need to have the time and incentive to assess fracture risk and explain the purpose of treatment to their patients. Secondary prevention, in which patients are identified for treatment on the basis of a previous low trauma fracture, is therefore the approach usually taken.

Several methods have been explored to enable fracture risk assessment and initiation of appropriate treatment—some based upon staff, others on IT and others upon a combination of the two. The multi-disciplinary Fracture Liaison Service (FLS) is one of the most successful of these systems [47, 48], incorporating rheumatologists, orthogeriatricians, other physicians, clinical nurse specialists and allied health professionals. Members of the FLS multidisciplinary team, coordinated by a lead clinician, work together to optimise the medical management of patients admitted with fracture, both in hospital and for long-term fracture prevention. [49•]. “Capture the Fracture®”, an initiative instituted by the International Osteoporosis Foundation (http://www.capturethefracture.org/), is “a global campaign to facilitate the implementation of coordinated, multi-disciplinary models of care for secondary fracture prevention.” Capture the Fracture has provided secondary fracture prevention guidance, and also a global map of secondary fracture prevention services, with a quality grading scheme, graded by assessed application and description of the service [50, 51]. This scheme has helped to document the huge variation in the quality, scope and availability of secondary prevention facilities, not only within but also between countries. The Capture the Fracture initiative aims to raise the quality and coverage of these services and has been shown to be both clinically valuable and cost-effective [52].

Vertebral fracture case finding is an additive approach to secondary fracture prevention as many such events go undetected—it has been shown that around 12% of postmenopausal women with osteoporosis have one or more vertebral deformities, but fewer than one in three of these individuals come to clinical attention [53]. Primary care-based screening strategies [54] and history taking methods distinguishing “vertebral fracture-type back pain” from other types of back pain may assist their detection [55]. Different methods for radiological assessment of vertebral fractures exist, including radiographs, CT scans and vertebral fracture software in DXA. The automated detection of prevalent vertebral fracture on CT scans using artificial intelligence technologies will be another avenue for secondary fracture prevention [56, 57].

### Primary Prevention: Starting Treatment in Individuals at High Fracture Risk

In osteoporosis, there is an ongoing debate regarding the benefits of a widespread systematic screening approach, leading to higher treatment rates (with its associated cost and side effect risk), and a case finding approach focused on those at highest individual risk (with its associated issue of under-treatment). Whilst DXA-based osteoporosis screening is officially a standard policy in the USA (at age 65 years in women, age 70 in men, and in individuals over 50 years who have suffered a fracture as an adult) [58], in the majority of countries, population screening is not judged to be cost-effective. Primary prevention is therefore generally focused on case finding strategies, reliant on the physician identifying clinical risk factors [15–17, 59].

**Fig. 3:**
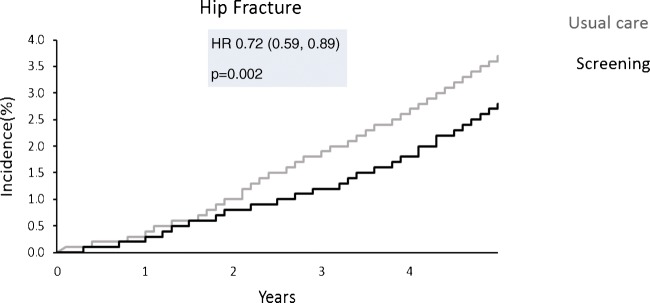
Cumulative incidence of hip fracture in the screening versus control arm in the SCOOP Trial. Produced with permission using data from Shepstone et al., 20198 [61••]

In the UK, a randomised controlled trial across seven centres was recently undertaken (the UK SCOOP study), examining both the clinical and cost-effectiveness of screening older women in primary care for primary fracture prevention. Around 12,500 older women were randomised to either screening and subsequent treatment (stratified using FRAX hip fracture probability) or usual care. The screening intervention was shown to lead to reduction in hip fracture risk by 28% (Fig. 3) [60, 61••]. Those at highest baseline fracture risk appeared to benefit most from screening (as would be expected, since these were the individuals targeted for treatment) [62], and importantly, were shown to be cost-effective (the cost per quality adjusted life year (QALY) gained was £2772 compared with the control arm) [63]. The finding that women who were identified by FRAX as moderate or high risk of fracture benefited most from a screening programme was supported by the Danish risk-stratified osteoporosis strategy evaluation (ROSE) study, though this study found no overall effect on fracture incidence of a screening strategy [64]. A recent evidence report and systematic review for the US Preventive Services Task Force concluded that screening to prevent osteoporosis in women may reduce hip fractures [65].

Once a patient has been identified as requiring fracture risk assessment, the threshold at which treatment should be given will vary according to factors such as healthcare provision, willingness to pay and cost of medications [5]. The majority of guidelines internationally use FRAX as the arbiter of fracture risk, but 38 of the 120 guidelines identified in a recent systematic review gave no direction on translating FRAX probabilities into a treatment decision [23]. Threshold setting is as much a philosophical as scientific process, which decisions around whether a level should be fixed, or age dependent, and calibrated to the specific country. Given the marked variation in fracture rates between countries, this latter consideration seems mandatory, and the benefits and caveats associated with fixed or age-dependent thresholds are presented in detail in [23]. In the UK, FRAX is linked to the age-dependent (up to the age of 70 years) thresholds of the National Osteoporosis Guideline Group (NOGG) [17], with the threshold predicated on the probability of future fracture conferred by a prior fracture. This approach has been shown to be cost effective in the UK, and contrasts markedly with that of the UK National Institute of Health and Care Excellence (NICE). In the 2017 technology appraisal of bisphosphonates [66], a pure health economic approach, in the context of a very common disease and extremely inexpensive therapies, led to a 1% risk of major osteoporotic fracture over 10 years as the threshold above which these medications were considered cost-effective. Unfortunately this was often interpreted as payers as an intervention threshold, a situation which if permitted to continue would have resulted in many adults at low risk of fracture being inappropriately treated [67], but which was later resolved by referral to NOGG guidance for clinical, rather than health economic thresholds [68].

## Conclusion

Recent decades have seen a dramatic transformation in osteoporosis, from having been historically viewed as an inescapable result of ageing, to now being a well-characterised chronic non-communicable disease, with diagnostic criteria, well-established methods of risk assessment and an enviable range of therapeutic medications. Despite this backdrop, however, there is evidence from the UK, USA and continental Europe that treatment rates have declined substantially in the last 5 years. With ageing populations and overstretched health services, osteoporosis may often fall off the bottom of the list of priorities for both clinicians and patients. The rare adverse effects of anti-resorptive therapies have become a disproportionately (and inappropriately) major concern, amplified by sensationalised media reports, which have usually been inadequately countered by the clinical academic community. These fears and the resulting reduced prescribing have been exacerbated by reductions in reimbursement in the USA, mirrored in new guidance. It is apparent that many patients, doctors and dentists and patients now appear more concerned by the rare but serious side effects of anti-resorptives than they are of the osteoporosis and fragility fractures.

The clear imperative to urgently tackle this issue has been recognised by key organisations such as the International Osteoporosis Foundation and the American Society for Bone and Mineral Research, leading to the publication of recommendations and roadmaps to address the critical care gap in osteoporosis treatment [69••, 70••]. Improved public awareness and public health strategies to improve bone health from a young age will also contribute to prevention of osteoporosis in future generations. Given the rapid ageing of the global population and the importance of good musculoskeletal health in old age, we must come together to ensure that during the coming decade, 2020–2030, hailed by the WHO and others as the “Decade of Healthy Ageing”, bone health and fracture prevention become the priority they so urgently need to be.
